# Empagliflozin Ammeliorates High Glucose Induced-Cardiac Dysfuntion in Human iPSC-Derived Cardiomyocytes

**DOI:** 10.1038/s41598-018-33293-2

**Published:** 2018-10-05

**Authors:** Kwong-Man Ng, Yee-Man Lau, Vidhu Dhandhania, Zhu-Jun Cai, Yee-Ki Lee, Wing-Hon Lai, Hung-Fat Tse, Chung-Wah Siu

**Affiliations:** Cardiology Division, Department of Medicine, Li Ka Shing Faculty of Medicine, the University of Hong Kong, Hong Kong SAR, Hong Kong China

## Abstract

Empagliflozin, a sodium-glucose co-transporter (SGLT) inhibitor, reduces heart failure and sudden cardiac death but the underlying mechanisms remain elusive. In cardiomyocytes, *SGLT1 and SGLT2* expression is upregulated in diabetes mellitus, heart failure, and myocardial infarction. We hypothesise that empagliflozin exerts direct effects on cardiomyocytes that attenuate diabetic cardiomyopathy. To test this hypothesis, cardiomyocytes derived from human induced pluripotent stem cells (hiPSCs) were used to test the potential effects of empagliflozin on neutralization of cardiac dysfunction induced by diabetic-like cultures. Our results indicated that insulin-free high glucose culture significantly increased the size of and *NPPB*, *SGLT1* and *SGLT2* expression of hiPSC-derived cardiomyocytes. In addition, high glucose-treated hiPSC-derived cardiomyocytes exhibited reduced contractility regardless of the increased calcium transient capacity. Interestingly, application of empagliflozin before or after high glucose treatment effectively reduced the high glucose-induced cardiac abnormalities. Since application of empagliflozin did not significantly alter viability or glycolytic capacity of the hiPSC-derived cardiomyocytes, it is plausible that empagliflozin exerts its effects via the down-regulation of *SGLT1*, *SGLT2* and *GLUT1* expression. These observations provide supportive evidence that may help explain its unexpected benefit observed in the EMPA-REG trial.

## Introduction

Empagliflozin is an inhibitor of the sodium–glucose co-transporter (SGLT) and improves glycemic control by increasing urinary glucose excretion. The unexpected results of the EMPA-REG trial^1^ revealed that it reduced the primary composite outcome of cardiovascular death, non-fatal myocardial infarction and non-fatal stroke (3-point major adverse cardiovascular events), cardiovascular death, hospitalization for heart failure, and overall mortality in patients with type 2 diabetes mellitus at high cardiovascular risk. The trial results were enthusiastically received because of the potential to substantially improve the prognosis of patients with diabetes mellitus. Nonetheless the mechanisms underlying these clinical benefits remain largely unexplained. Recent studies have demonstrated the direct cardiac effects of empagliflozin in rodent and rabbit models^2,3^. For example, Baartscheer *et al*. demonstrated that application of empagliflozin could reduce the effects of high glucose-induced increased intracellular calcium and sodium levels in ventricular myocytes isolated from rat and rabbit^2^. In addition, Steven *et al*. demonstrated the beneficial effects of empagliflozin in improving primary diabetic complications in ZDF rats^3^. Nonetheless due to the substantial physiological differences between human and rodent hearts^2^, it remains unclear whether human cardiomyocytes will respond to empagliflozin in a similar manner.

**Figure 1:**
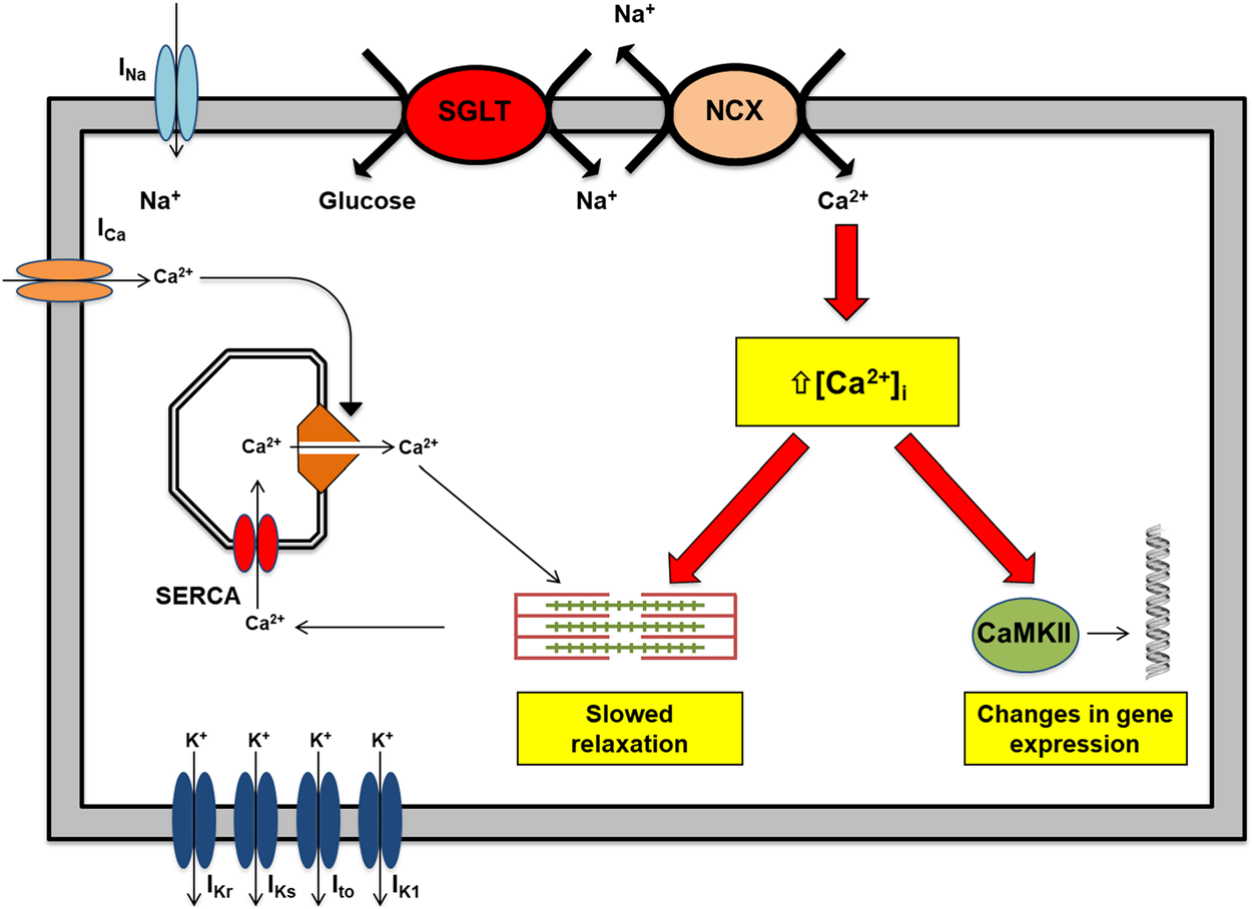
Schematic representation of the proposed mechanisms underlying diabetic cardiomyopathy in relation to sodium-glucose co-transporter (SGLT). Upregulation of *SGLT* in cardiomyocytes in the presence of diabetes mellitus results in an increase in sodium influx into cardiomyocytes that in turn increases cytosolic calcium loading via the sodium-calcium exchanger (NCX). Intracellular calcium overload can lead to (1) delayed after depolarizations (DADs), triggers for tachycardia, (2) impaired excitation-contraction coupling, and (3) activation of calcium-sensitive signaling pathways, leading to pathological changes.

We hypothesise that empagliflozin exerts direct effects on cardiomyocytes in the presence of diabetes mellitus that favorably alter their cellular functions, leading to the observed benefit in the EMPA-REG trial^1^. Our proposed hypothesis is based on several well-established facts. First, although the primary endpoint of the EMPA-REG trial^1^ was a composite of non-fatal myocardial infarct, non-fatal stroke, and cardiovascular mortality that was reduced by 14%, it was entirely driven by a substantial reduction in cardiovascular death unrelated to vascular events such as myocardial infarction or stroke. This raises the possibility that the drug confers its clinical benefits through non-atherothrombotic mechanisms. Indeed, the favorable but small differences in cardiometabolic factors such as HbA1c, blood pressure, or weight loss achieved with empagliflozin cannot be attributed to such a magnitude of improvement in cardiovascular outcomes. In concordance with this notion, the very early separation of cumulative incidence curves for cardiovascular mortality and heart failure-related hospitalization within a few weeks of randomization likewise could not be explained by the modification of atherosclerosis progression. Second, although the primary site of action of SGLT is in the proximal tubules of nephrons, *SGLT1* expression has been previously reported in healthy human myocardium suggesting a possible physiological function. More importantly, myocardial expression of *SGLT1* is up-regulated in type II diabetes mellitus, myocardial ischemia, and severe heart failure^4^. This up-regulation in these conditions can theoretically increase sodium as well as glucose entry into cardiomyocytes. Through the reverse mode of sodium-calcium exchanger (NCX), the increased intracellular sodium in these conditions in turn increases cytosolic calcium. (Fig. 1) Calcium signaling is central to cardiac physiology, from excitation-contraction coupling and relaxation mechanisms to calcium-dependent transcriptional regulation via multifunctional calcium-calmodulin-dependent protein kinase II (CaMKII)^5^. Intracellular calcium overload in cardiomyocytes impairs excitation-contraction coupling and relaxation mechanisms, leads to electrical instability, and activates a calcium sensitive hypertrophic signaling pathway. Blockade of SGLT with empagliflozin can attenuate these pathological processes with the consequent clinical benefits observed in the EMPA-REG trial^1^. Theoretically, direct functional characterization of human cardiomyocytes obtained from patients with diabetic cardiomyopathy before and after empagliflozin treatment will provide much-needed insight, but is limited by obvious ethical concerns and technical difficulties. To provide experimental data to support our hypothesis, we established a chemically-induced *in vitro* model of diabetic cardiomyopathy using human induced pluripotent stem cell (hiPSC)-derived cardiomyocytes to elucidate the pharmacological effects of empagliflozin. In the model, empagliflozin abolished hyperglycemia-induced hypertrophic changes, alleviated the abnormal calcium-handling, and restored contractility of high glucose (HG)-treated hiPSC-derived cardiomyocytes.

## Results

### High Glucose Induced Hypertrophic Changes in hiPSC-derived cardiomyocytes

**Figure 2:**
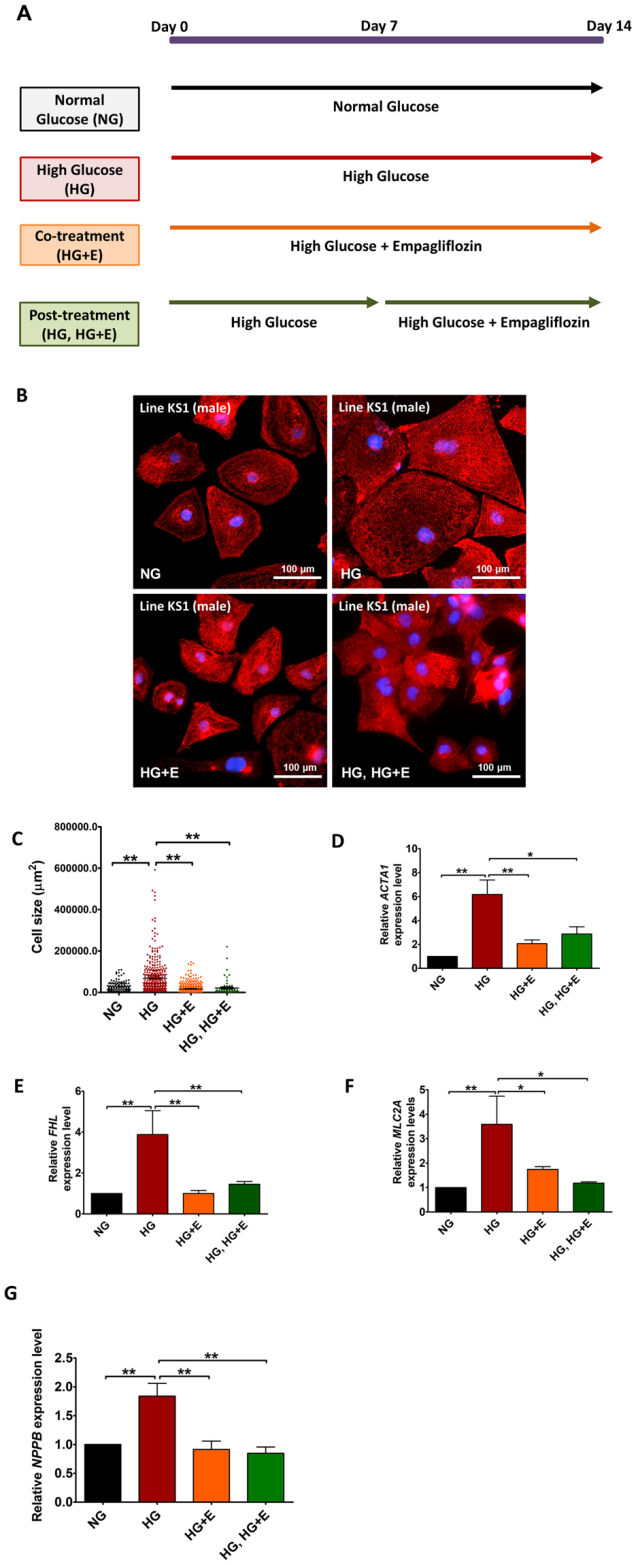
Hypertrophic Changes under High Glucose Condition and Empagliflozin to hiPSC-derived cardiomyocytes. (**A**) Schematic outline of the time and duration of high glucose treatment and empagliflozin application. (**B**) Representative immunofluorescence staining of KS1 hiPSC-derived cardiomyocytes cultured in normal glucose (5.5 mM); high glucose (22 mM); high glucose and empagliflozin; and high glucose for 7 days followed by empagliflozin (Red: α-Actinin; blue: DAPI); **(C)** The cell size; **(D**–**G)** Relative expressions of *ACTA1*, *FHL*, *MLC2A* and *NPPB* respectively. Abbreviations: NG: normal glucose; HG: high glucose; HG + E: high glucose and empagliflozin; and HG, HG + E: high glucose for 7 days followed with empagliflozin. *p < 0.05; **p < 0.01.

To recreate the diabetogenic environment, hiPSC (Line KS1)-derived cardiomyocytes were exposed to a high glucose (HG) environment by supplementing standard culture medium with 22 mM glucose for 14 days (Fig. 2A). Figure 2B shows the immunofluorescence staining of cardiomyocytes with normal glucose (NG) (5.5 mM) and HG culture medium. HG-treated cardiomyocytes were 5-fold larger than NG-treated cardiomyocytes (mean cell size: 67,163 ± 4,370 µm^2^ *vs*. 13,321 ± 624 µm^2^, *p* < 0.001; median cell size: 37,084 µm^2^ *vs*. 9,057 µm^2^, *p* < 0.001) (Fig. 2C). In line with the observed hypertrophic changes, high glucose treatment significantly increased the expression of the cardiac hypertrophy markers *ACTA1* (HG vs NG: about 6.0 fold, p < 0.01), *FHL* (HG vs NG: about 3.9 fold, p < 0.01) and *MLC2A* (HG vs NG: about 3.5 fold, p < 0.01) (Fig. 2D–F). In addition, exposure to a HG environment increased the expression of *NPPB*, which encodes the brain-type natriuretic peptide (BNP), in hiPSC-derived cardiomyocytes (Fig. 2G).

### Effects of High Glucose on Contractility and Calcium homeostasis of hiPSC-derived cardiomyocytes

**Figure 3:**
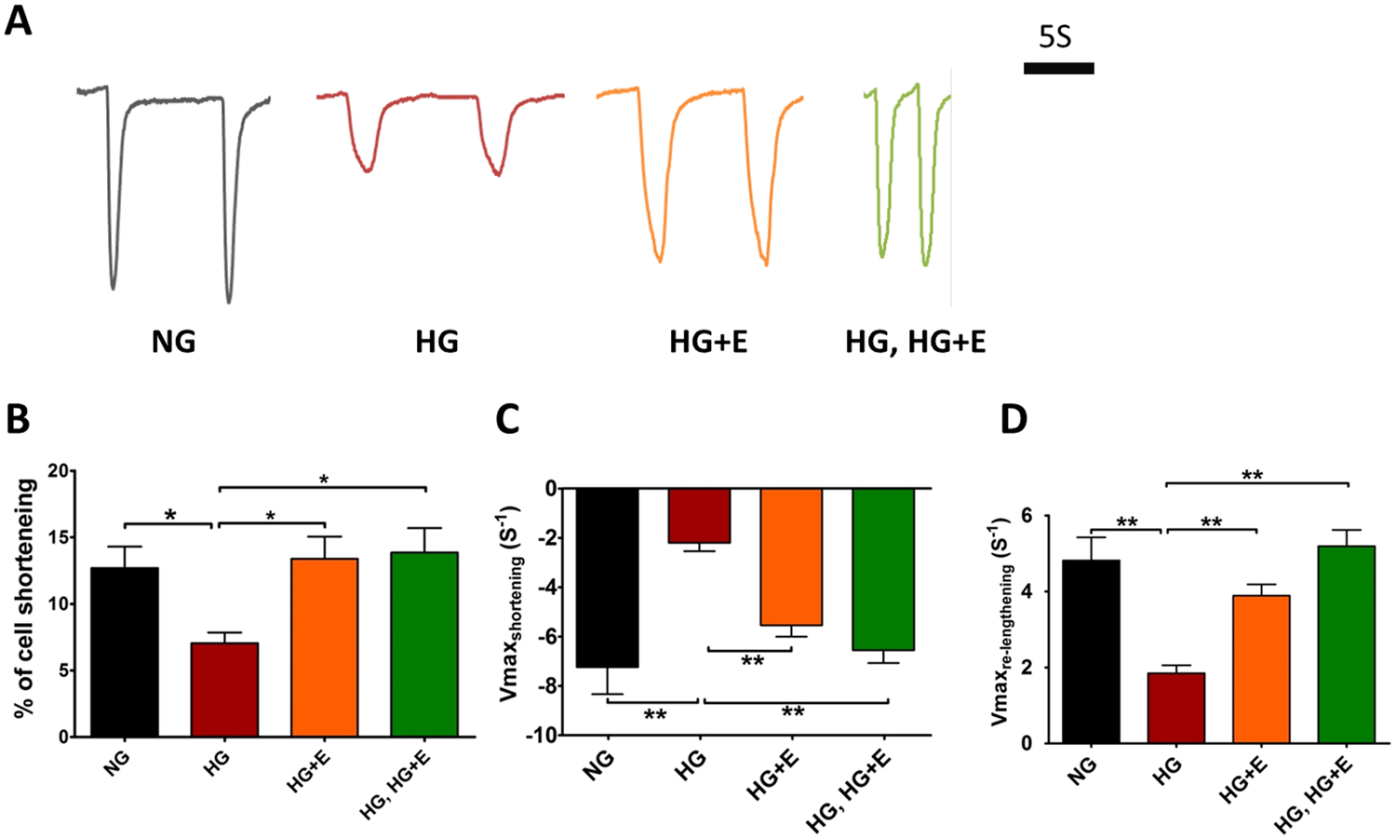
Contractility of hiPSC-cardiomyocytes under High Glucose Condition and Empagliflozin. (**A**) Representative tracings of cell changes. **(B)** Percentage cell shortening; **(C)** Maximal velocity of cell shortening and **(D)** Maximal velocity of cell re-lengthening. Abbreviations: NG: normal glucose; HG: high glucose; HG + E: high glucose and empagliflozin; and HG, HG + E: high glucose for 7 days followed with empagliflozin. *p < 0.05; **p < 0.01.

**Figure 4:**
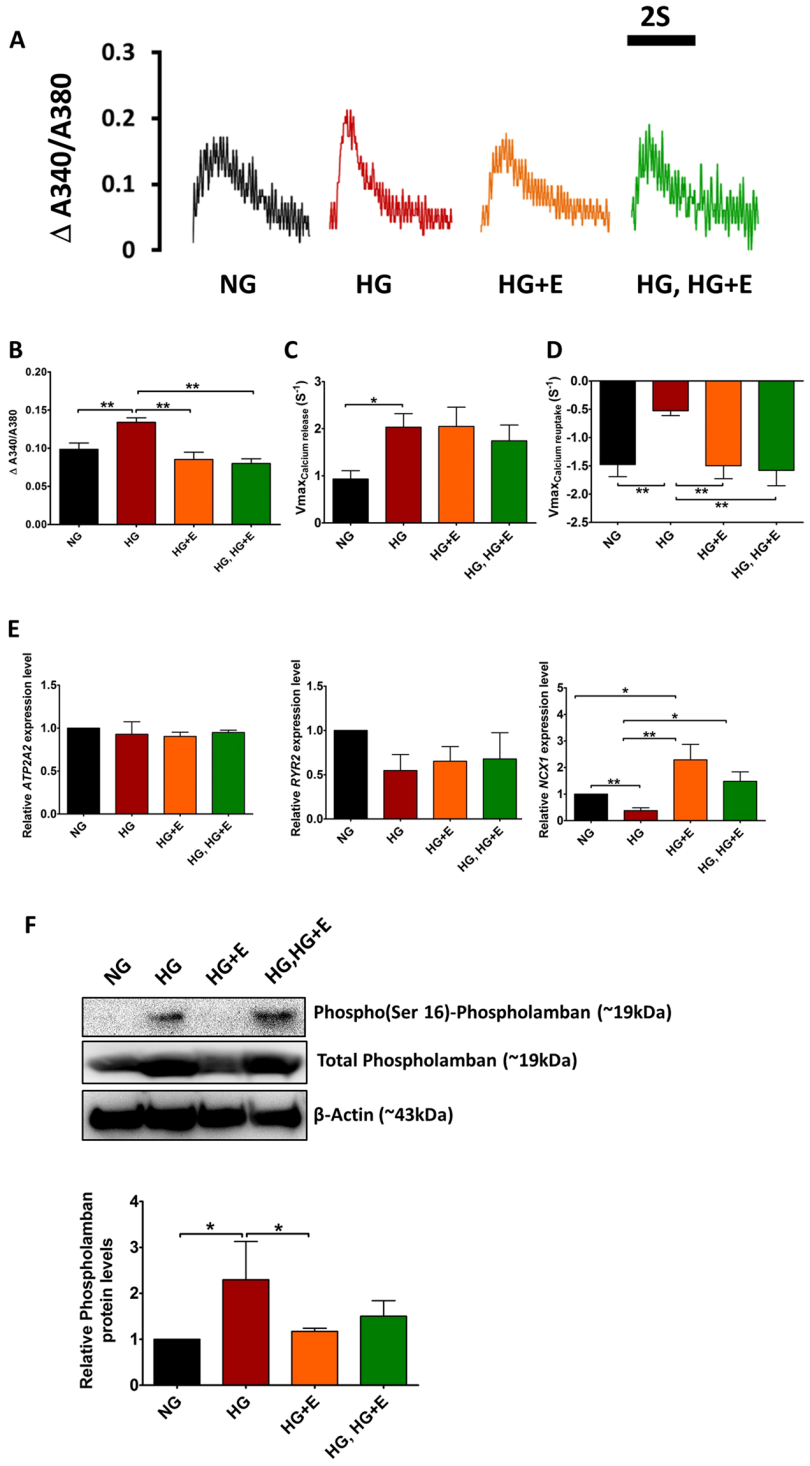
Calcium Handling Properties of hiPSC-derived cardiomyocytes under High Glucose Condition and Empagliflozin. (**A**) Representative tracings of whole-cell calcium transients in hiPSC-derived cardiomyocytes; **(B)** Amplitudes of whole-cell calcium transients; **(C)** Maximal upstroke velocity of whole-cell calcium transients; and **(D)** Maximal decay velocity of whole-cell calcium transients. Abbreviations: NG: normal glucose; HG: high glucose; HG + E: high glucose and empagliflozin; and HG, HG + E: high glucose for 7 days followed with empagliflozin. **(E)** The expression of *ATP2A2*, *RYR2* and *NCX1* was evaluated by quantitative PCR analysis using the expression of *TNNT2* as an internal control. Abbreviations: NG: normal glucose; HG: high glucose; HG + E: high glucose and empagliflozin; and HG, HG + E: high glucose for 7 days followed with empagliflozin. **(F)** Protein level of phospho-phospholamban and total phospholamban was evaluated by Western blot analysis, the protein level of β-actin was used as internal reference. *p < 0.05 and **p < 0.01. For the full-length image of gels and blots shown in this figure, please refer to Supplementary Figs S4 and S5.

High glucose treatment did not significantly alter the beating rate of the hiPSCs-derived cardiomyocytes (Supplementary Fig. S2). Nonetheless in contrast to the hypertrophic changes, a video-based edge detection system revealed that HG-treated cardiomyocytes exhibited a markedly reduced contractility, measured as the percentage of peak cell shortening during field stimulation (from 12.7 ± 1.6% to 7.1 ± 0.8%, 44% reduction, *p* < 0.05), and reduced maximal velocity of shortening from −7.2 ± 1.1 s^−1^ to −2.2 ± 0.3 s^−1^ (*p* < 0.05) (Fig. 3). The maximal velocity of re-lengthening also reduced from 4.8 ± 0.6 m/sec to 1.9 ± 0.2 m/sec (*p* < 0.05) (Fig. 3). In addition, despite the overall larger whole-cell calcium transients in HG-treated cardiomyocytes (0.13 ± 0.01, *vs*. 0.10 ± 0.01, *p* < 0.05), and faster maximal upstroke velocity (2.03 ± 0.29 s^−1^, vs. 0.93 ± 0.18 s^−1^, *p* < 0.05) compared with NG-treated cardiomyocytes, the maximal decay velocity was significantly retarded (−0.52 ± 0.08 s^−1^, vs. −1.48 ± 0.21 s^−1^, *p* < 0.05) (Fig. 4). These results suggest that coupling of the calcium transient and contraction was less effective in cardiomyocytes treated with high glucose, because the increased calcium handling capacity did not result in increased contractility. In addition, although we could find no significant differences in the expression of *ATP2A2* or *RYR2* between control and HG-treated cardiomyocytes, *NCX1* expression was significantly reduced in the latter (about 60% reduction, *p* < 0.01) (Fig. 4E). Protein level of total and phosphorylated phospholamban was also markedly increased in the HG-treated cardiomyocytes (Fig. 4F).

### Effects of Empagliflozin on HG-induced Changes

To test the hypothesis that empagliflozin can alleviate diabetic cardiomyopathy, empagliflozin (5 μM) was administrated to hiPSC-derived cardiomyocytes at the same time as HG treatment (Co-treatment, HG + E), and 7 days later (post-treatment, HG, HG + E). With this concentration of empagliflozin, no significant cell death was observed (for detail, see Supplementary Fig. S1). As indicated in Fig. 2, application of empagliflozin, either co-treatment or post-treatment, almost completely abolished the hypertrophic changes in HG-treated cardiomyocytes. The mean and median size of HG-treated cardiomyocytes reduced from 67,163 ± 4,370 µm^2^ and 37,084 µm^2^ respectively, to 16,687 ± 17,578 µm^2^ and 11,738 µm^2^ in HG + E-treated cardiomyocytes (*p* < 0.05), and to 21,339 ± 35,942 µm^2^ *vs*. 9,408 µm^2^ in HG, HG + E-treated cardiomyocytes (*p* < 0.05). Size of empagliflozin-treated cardiomyocytes was not significantly different to that of cardiomyocytes in NG culture conditions (Fig. 2B,C). Interestingly, empagliflozin normalized the elevated *ACTA1*, *FHL*,*MLC2A and NPPB* expressions in HG-treated cardiomyocytes (Fig. 2D–G).

Regarding the contractile functions, HG-treated cardiomyocytes exhibited a marked decrease in the percentage of cell shortening (contractility) compared with NG-treated cardiomyocytes (NG *vs* HG: 13.4 ± 1.7%, *vs*. 7.1 ± 0.8%, *p* < 0.05) (Fig. 3A,B). Similar changes were also observed in the rates of cell shortening and re-lengthening (Fig. 3C,D). Empagliflozin treatment, regardless of the time of application (HG + E and HG, HG + E groups), abolished such effects of high glucose treatment. For example, HG + E-treated cardiomyocytes showed an improved maximal velocity of shortening from −2.2 ± 0.3 s^−1^ to −5.5 ± 0.5 s^−1^ (HG *vs* HG + E, *p* < 0.05) and maximal velocity of re-lengthening from 1.9 ± 0.2 m/sec to 3.9 ± 0.3 m/sec (HG *vs* HG + E, *p* < 0.05) (Fig. 3C,D). Similar trends were also observed in HG, HG + E-treated cardiomyocytes. In addition, empagliflozin treatment regardless of the timing of application normalized the amplitude of whole-cell calcium transients and the maximal decay velocity in cardiomyocytes and they became similar to those of NG-treated cardiomyocytes (Fig. 4). Interestingly, although empagliflozin treatment did not significantly alter *ATP2A2* or *RYR2* expression, *NXC1* expression was significantly upregulated in both HG + E and HG, HG + E treated cardiomyocytes (Fig. 4E). In terms of the protein level of phospholamban, although simultaneous application of empagliflozin (HG + E) could abolish the HG-induced increases in the total and phosphorylated phospholamban level, later application (HG, HG + E) of the drug appeared to be non-effective (Fig. 4F).

### Effects of Empagliflozin on expression of multiple glucose transporter genes

**Figure 5:**
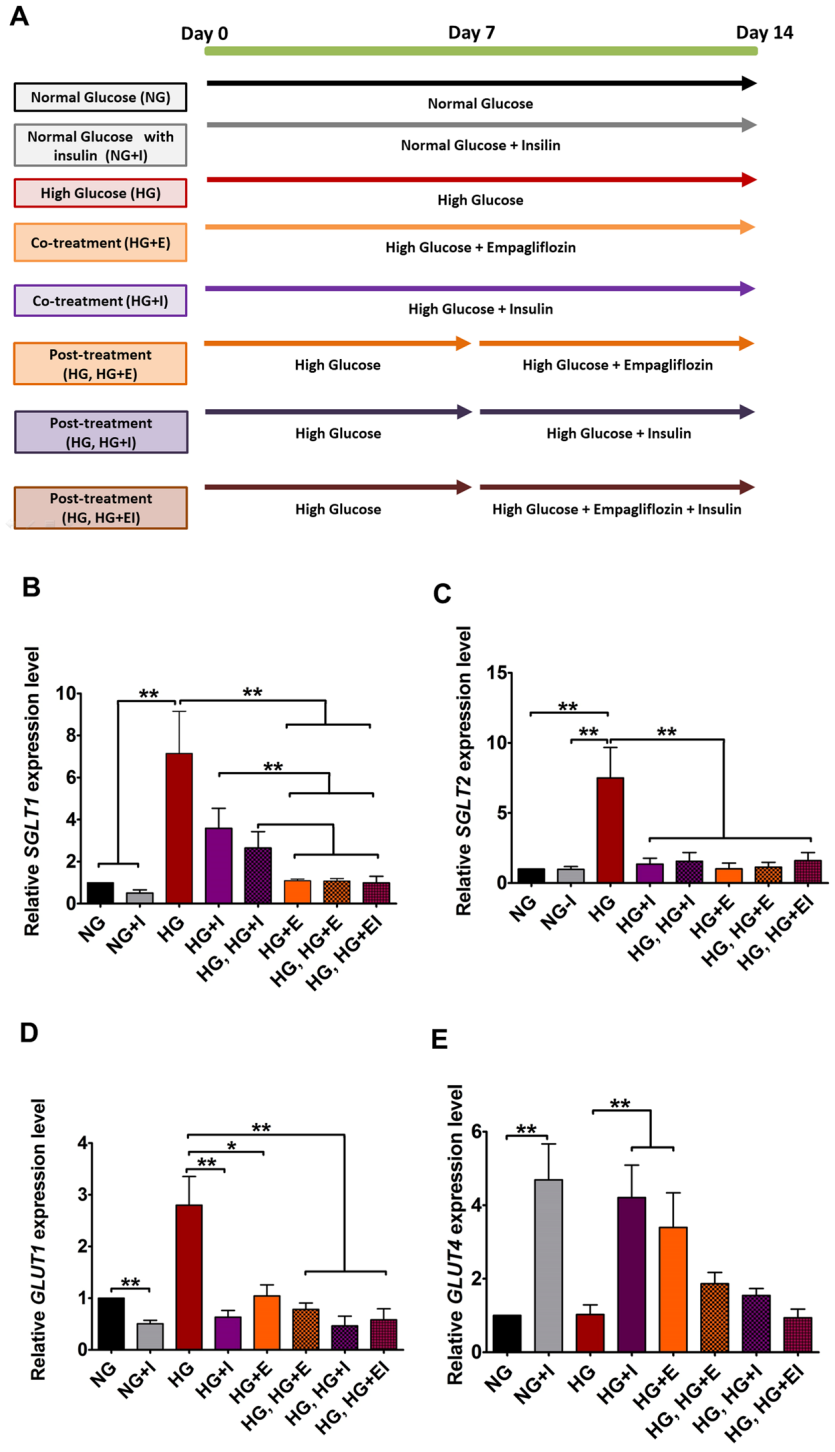
Effects of High Glucose, Empagliflozin and insulin on the expression of glucose transporters in hiPSC-derived Cardiomyocytes. (**A**) Schematic outline of the times and durations of high glucose treatment and the application of empagliflozin and insulin. (**B**,**C**) Relative expressions of *SGLT1* and *SGLT2*. (**D** and **E**) Relative expressions of *GLUT1* and *GLUT4*. *p < 0.05 and **p < 0.01.

To deduce the mechanism underlying the anti-glucose effects of empagliflozin, we further evaluated the expression of various glucose transporter genes that are likely to be responsive to empagliflozin or insulin (Fig. 5). Figure 5A outlines the treatment scheme. As indicated in Fig. 5B,C, HG-treatment significantly elevated the expression of *SGLT1* (~7.1 folds, *p* < 0.01) and *SGLT2* (~7.5 folds, *p* < 0.01). Regardless of the time of application or the presence of insulin, empagliflozin treatment restored *SGLT1* and *SGLT2* expression to the control level. In addition to *SGLT1* and *SGLT2*, we also evaluated the expression of *GLUT1* and *GLUT4* that encode the glucose-regulated glucose transporter 1 and the insulin-responsive glucose transporter 4 respectively. As shown in Fig. 5D, prolonged high glucose treatment (HG) significantly increased *GLUT1* expression (~2.8 folds, *p* < 0.01), while application of empagliflozin or insulin abolished this change. On the contrary, although there was no significant change in *GLUT4* expression in HG-treated cardiomyocytes, early application of empagliflozin (HG + E) or insulin (HG + I) significantly up-regulated its expression (Fig. 5E). Interestingly, the effects of both empagliflozin and insulin on *GLUT4* were not obvious when they were applied 7 days after the high glucose treatment. This suggests that high glucose treatment renders cardiomyocytes immune to empagliflozin and insulin.

**Figure 6:**
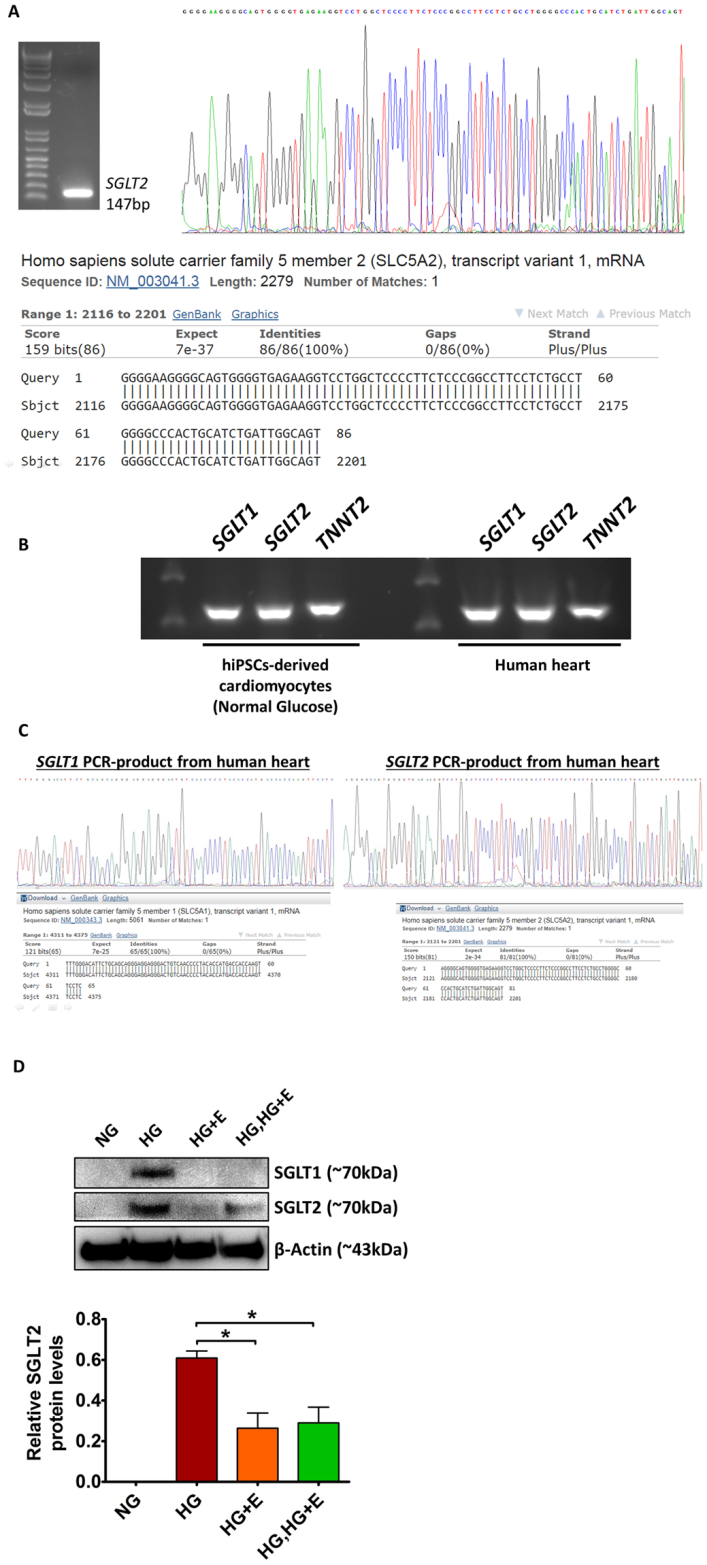
Expression of *SGLT1* and *SGLT2* in hiPSC-derived cardiomyocytes and human heart tissue. (**A**) Confirmation of the identity of the PCR product obtained from *SGLT2*-specific amplifications by DNA sequencing analysis. **(B**,**C)** Expression of *SGLT1* and *SGLT2* in human heart tissue were confirmed by PCR analysis and DNA sequencing analysis. **(D)** Protein levels of *SGLT1* and *SGLT2* in the hiPSC-derived cardiomyocytes were evaluated by Western blot analysis. *p < *0*.*05*. For the full-length images of gels and blots shown in this figure, please refer to Supplementary Figs S4 and S5.

Based on the controversy surrounding the cardiac expression of *SGLT2*^6,7^, we have further confirmed the identity of the *SGLT2* PCR products by sequencing analysis (Fig. 6A). We have also demonstrated that *SGLT1* and *SGLT2* transcripts are present in human heart tissue (Fig. 6B,C). To provide additional evidence of the effects of high glucose and empagliflozin on *SGLT1* and *SGLT2* expression, we also evaluated the *SGLT1* and *SGLT2* protein levels by Western blot analysis. As shown in Fig. 6D, for *SGLT1*, we were able to detect this protein only in the high glucose treated group. On the contrary, for *SGLT2*, we were able to detect the *SGLT2* proteins in all high glucose treated groups but not the normal glucose group. We believe this was due to the much lower sensitivity of the Western Blot analysis compared with real-time PCR analysis. Although we were unable to detect the immuno-reactive *SGLT1* and *SGLT2* in certain groups, the results are in line with the PCR analysis that high glucose treatment increased the level of *SGLT1* and 2, while empagliflozin treatment helped reduce the effects of high glucose.

### Effects of High Glucose and Empagliflozin on protein level of Caspase 3 and 7

**Figure 7:**
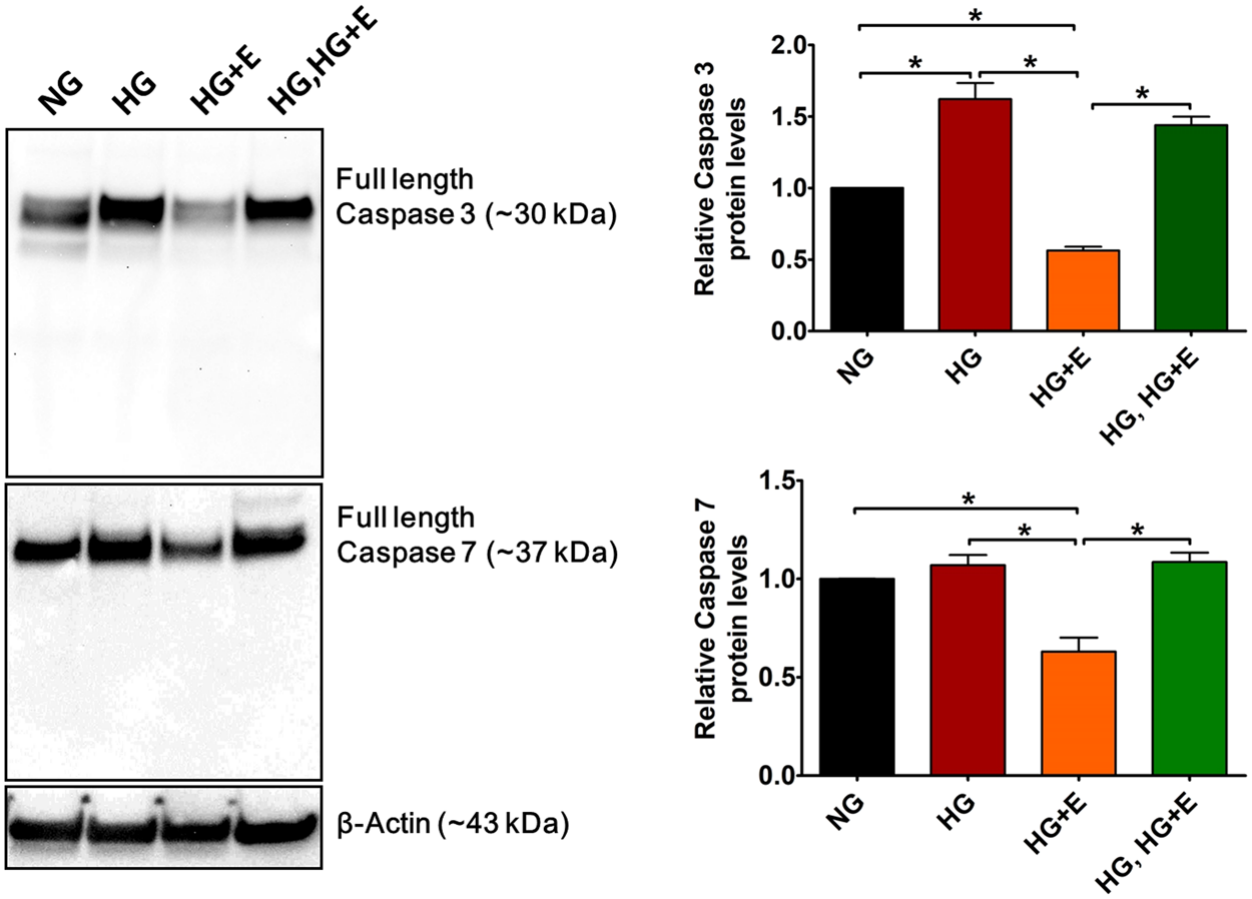
Effects of High glucose and empagliflozin on the protein level of caspase 3 and caspase 7. Protein level of caspase 3 and caspase 7 was evaluated by Western blot analysis using antibodies that could detect both full length and cleaved forms of the target protein. The protein level of β-actin was used as internal control for statistical analysis. *p < *0*.*05*. (n = 4).

High glucose-induced intracellular calcium overload promotes oxidative stress and ultimately leads to cell apoptosis^8^. Although no significant cell death was observed in any treatment group (data not shown), Western blot analysis revealed that high glucose treatment significantly increased caspase 3 protein level (HG vs NG, ~1.6 fold increase, p < 0.05). Interestingly, such change was abolished by co-treatment (HG + E), but not post-treatment (HG, HG + E) with empagliflozin (Fig. 7). High glucose treatment did not alter the protein level of caspase 7, although compared with the other three groups (NG, HG and HG, HG + E), a significantly reduced caspase 7 level was observed in the empagliflozin co-treatment groups (HG + E). It should be noted that in all samples, no cleave caspase 3 and 7 could be detected even when the experiments were repeated using antibodies specific to the cleaved forms (data not shown). These results suggest that although relatively short-term (14 days) high glucose treatment was not sufficient to induce significant apoptosis, such treatment may direct the cardiomyocytes to early apoptotic stages.

### Effects of high glucose and empagliflozin on the cardiac function of cardiomyocytes derived from different human iPSC-lines

**Figure 8:**
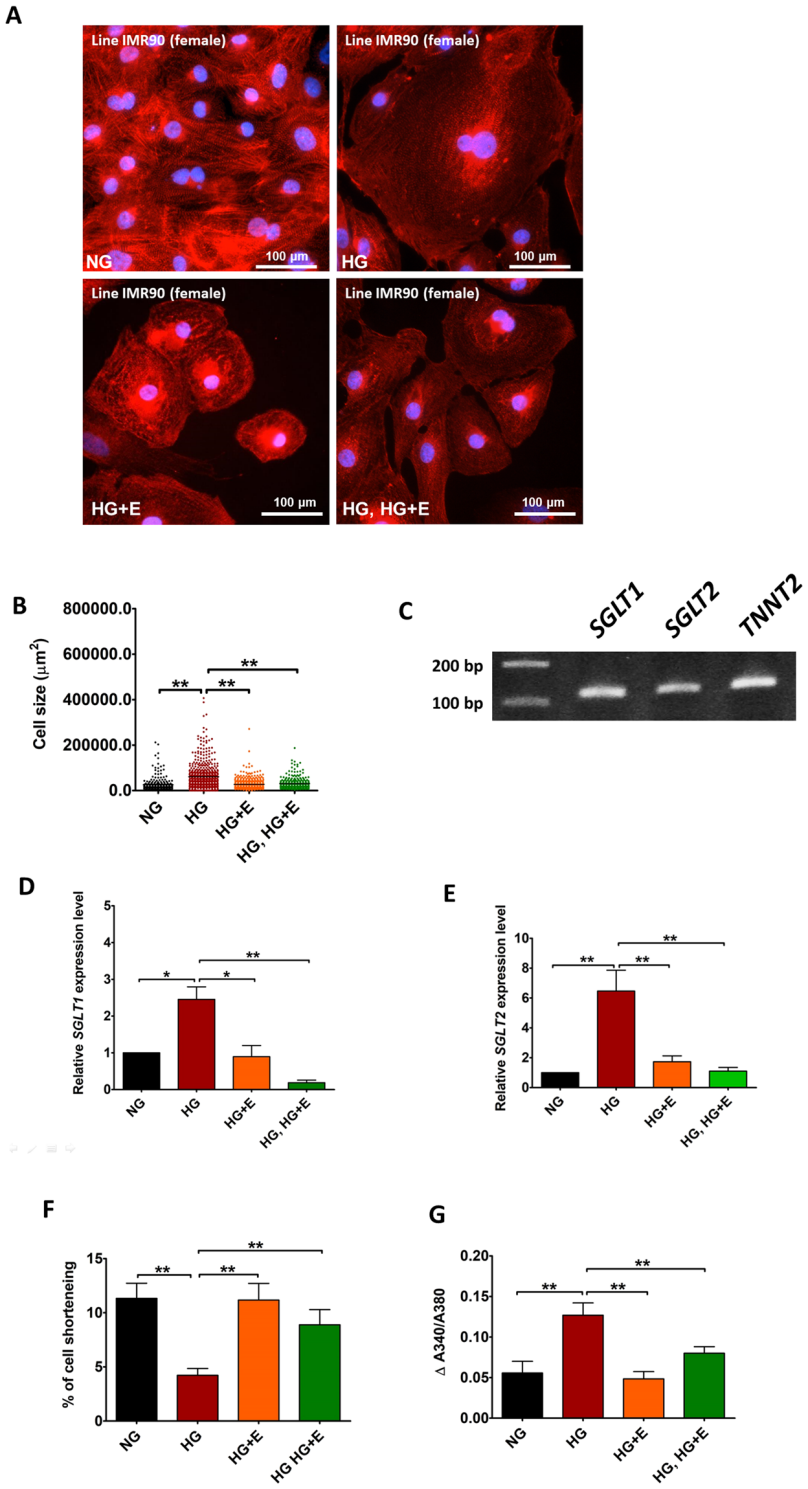
Effects of High glucose and empagliflozin on the functional alterations of cardiomyocytes derived from IMR90 hiPSCs. (**A**) Representative immunofluorescence staining, and (**B**) cell size of IMR90 hiPSC-derived cardiomyocytes cultured in normal glucose (5.5 mM); high glucose (22 mM); high glucose and empagliflozin; and high glucose for 7 days followed by empagliflozin (Red: α-Actinin; blue: DAPI). (**C**) Existence of *SGLT1* and *SGLT2* transcripts in the IMR90 hiPSC-derived cardiomyocytes were evaluated by PCR analysis. (**D**,**E**) Real-time quantitative PCR analysis of *SGLT1* and *SGLT2* expression. (**F**) % of cell shortening. (**G**) Amplitude of intracellular calcium trainsets. *p < *0*.*05*; **P < *0*.*01*.

To confirm that the effects of high glucose and empagliflozin were not confined to a particular cell line, we repeated specific experiments on cardiomyocytes derived from another individual (Line IMR90, derived from normal female). As shown in Fig. 8A,B, high glucose treatment significantly increased the size of IMR90-iPSC-derived cardiomyocytes, while empagliflozin, regardless of the time of application, abolished such effect. After confirming the expression of *SGLT 1* and 2 in the IMR90-iPSCs by conventional PCR analysis (Fig. 8C), real-time quantitative PCR analysis was used to evaluate the changes to *SGLT1* and *SGLT*2 expression upon high glucose and empagliflozin treatment. As shown in Fig. 8D,E, high glucose treatment significantly increased *SGLT1* and *SGLT2* expression only in the absence of empagliflozin. Similar to the results obtained from the KS1-iPSC-derived cardiomyocytes, high glucose treated cardiomyocytes exhibited significantly decreased contractility but significantly increased amplitudes of calcium transient, and these changes were reduced in the presence of empagliflozin (Fig. 8F,G).

### Effects of High Glucose and Empagliflozin on Bioenergetics of hiPSC-Derived Cardiomyocytes

To determine both the glycolytic flux and glycolytic capacity of hiPSC-derived cardiomyocytes under NG-, HG-, HG + E- and HG, HG + E-treatment, ECAER, a surrogate for anaerobic glycolysis measured using a Seahorse XF^e^24 flux analyzer, was assayed consecutively following injection of glucose, oligomycin and 2-DG. As shown in Supplementary Fig. S3A, addition of 10 mM glucose to hiPSC-derived cardiomyocytes triggered a glycolytic flux of 4.16 ± 0.55mpH/min/µg in NG treated cardiomyocytes, 1.82 ± 0.26mpH/min/µg in HG-treated cardiomyocytes and 2.01 ± 0.41 mpH/min/µg in HG + E treated cardiomyocytes. The subsequent addition of oligomycin caused a further increase in ECAR to 6.42 ± 0.59mpH/min/µg, 3.32 ± 0.0.44 mpH/min/µg and 3.62 ± 0.97mpH/min/µg in NG-, HG- and HG + E- treated cardiomyocytes respectively, indicating an elevated glucose flux toward lactate and revealing the glycolytic capacity. The final addition of glycolysis inhibitor 2-DG abolished the overall glycolysis. Collectively, hiPSC-derived cardiomyocytes in NG culture conditions exhibited a more robust and sustained glycolytic capacity than HG-treated and HG + E-treated cardiomyocytes (***p < 0.001). More importantly, there was no significant difference between HG-treated and HG + E-treated cardiomyocytes. The calculated glycolytic flux and glycolytic capacity from the glycolysis experiment are shown in Supplementary Figs S3B and S3C.

## Discussion

For decades, it has been well known that patients with diabetes mellitus can develop ventricular dysfunction, even in the absence of coronary artery disease^9^. The condition is commonly referred to as diabetic cardiomyopathy and is considered a cardiac muscle disorder due to the metabolic consequences of diabetes mellitus^10^. It is characterized in the early stages by ventricular hypertrophy and diastolic dysfunction, and in later stages by systolic dysfunction that progresses to decompensated heart failure, resulting in morbidities and mortality^11^. The pathogenesis of diabetic cardiomyopathy is complex and multifactorial. Mechanisms that include impaired calcium handling, up-regulated renin-angiotensin system, increased oxidative stress, altered substrate metabolism, and mitochondrial dysfunction have been implicated^12^.

Previous study suggested that hiPSC-derived cardiomyocytes may be metabolically immature and unable to represent the diabetic cardiomyopathy phenotypes^13^. Nevertheless our results suggest that the use of high glucose and insulin free medium is sufficient to induce most of the characteristic phenotypes of diabetic cardiomyopathy, such as cardiac hypertrophy, increased NPPB expression and impaired contractile functions. According to our proposed hypothesis and experimental data, in the diabetic milieu, the high glucose environment together with the induced upregulation of myocardial *SGLT1*/*SGLT2* expression results in excessive sodium entry into cardiomyocytes. Through the reverse mode of *NCX*, the increased sodium entry into cardiomyocytes leads to an increase in cytosolic calcium and may play a central role in cardiac pathophysiology^14^ (Fig. 1). For instance, high intracellular calcium concentration in cardiomyocytes activates a number of calcium-sensitive signaling pathways in cardiomyocytes such as calcineurin–NFAT29 and CaMKII–histone deacetylase pathways, leading to pathological cardiac hypertrophy^5^. It has been recently demonstrated that high glucose culture leads to increased intracellular sodium and calcium levels in isolated mouse and rabbit cardiomyocytes^2^, and such changes can be attenuated by empagliflozin. Although Na^+^/Ca^2+^ exchanger is a bidirectional ion-channel, the increase in Na^+^/Ca^2+^ exchanger levels is expected to increase the capacity for Na^+^/Ca^2+^ influx and efflux and help cells to maintain ion homeostasis.

In addition, high glucose-induced intracellular calcium overload promotes oxidative stress and ultimately leads to cell apoptosis^8^. Although we saw no significant cell death in any treatment group during the course of study, high glucose treatment significantly increased caspase 3 protein levels. This suggests that high glucose treatment may predispose cardiomyocytes to an early apoptotic stage. Interestingly, such a change could be abolished by early-, but not post-, application of empagliflozin.

Furthermore, altered calcium homeostasis in cardiomyocytes can result in impaired excitation-contraction coupling and decreased contractility, a hallmark of diabetic cardiomyopathy^15^, as well as chronic heart failure. One of our most striking experimental results was the high glucose-induced hypertrophic change in hiPSC-derived cardiomyocytes with increased cell size, associated with increased *SGLT1*, *SGLT2* and *NPPB* expression that was exhibited by marked hypertrophic changes with increase in cell size, and increased *NPPB* and *SGLT1* expression resembling that seen in human diabetic cardiomyopathy. In addition, hiPSC-derived cardiomyocytes under diabetogenic conditions exhibited markedly impaired contractility with reduced cell shortening as well as relaxation and reduced re-lengthening velocity, despite the overall larger whole-cell calcium transients and a faster maximal upstroke velocity of calcium transients. It has been previously suggested that abnormal calcium handling with impaired sensitivity of myofibrils to calcium is the most pivotal mechanism that leads to the reduced contractility in diabetic cardiomyopathy^15^. On the contrary, in our experiment, hiPSC-derived cardiomyocytes under diabetogenic conditions also exhibited retarded decay velocity of calcium transient, indicating impaired calcium extrusion. At the whole-heart level, this could result in impaired left ventricular relaxation and increased diastolic wall tension. The consequent diastolic dysfunction impairs coronary blood flow to the already ischemic myocardium, further worsening pre-existing ischemia. Electro-physiologically, increased intracellular calcium concentration in cardiomyocytes destabilizes the cell membrane, leading to increased excitability such as delayed after-depolarizations, predisposing to arrhythmias and resulting in sudden arrhythmic death. In fact, the sodium entry and exit pathway and the consequential influx and efflux of calcium through the *NCX1* has been acknowledged to play a pivotal role in the pathogenesis of various heart diseases including cardiac arrhythmias and heart failure^16^. Indeed pharmacological inhibition of sodium entry to lower an abnormally high cytosolic calcium concentration has recently emerged as a new therapeutic target for such conditions^17^. In concordance with this, in our experiment, empagliflozin almost completely abolished these hypertrophic changes and normalized *NPPB* expression of hiPSC-derived cardiomyocytes. The drug also attenuated the altered calcium handing and restored contractility and relaxation of high-glucose treated cardiomyocytes, independent of the glycolytic capacity, within one week of administration. These direct myocardial effects of empagliflozin demonstrated in our experiments fit well with the early reduction of non-atherothrombotic cardiovascular events such as heart failure hospitalization and mortality in the EMPA-REG trial, supporting the hypothesis that *SGLT* inhibition improves the functionality of cardiomyocytes with consequent clinical benefits. Alongside this hypothesis, one would expect similar clinical benefits from other *SGLT* inhibitors, particularly those less selective for *SGLT2*. In a meta-analysis of 21 phase 2b-3 studies that involved 9,339 diabetic patients at varying cardiovascular risk treated with empagliflozin, there were also similar but statistically insignificant reductions in cardiovascular death and heart failure-related hospitalizations^18^. Nonetheless because of the low cardiovascular event rate in the study populations and the short study durations, these studies lack the statistical power to fully confirm the potential benefit of empagliflozin. An ongoing prospective, randomized cardiovascular outcomes trial DECLARE TIMI-58, with a larger sample size and a longer follow-up period, is expected to document the effects of empagliflozin on cardiovascular outcomes amongst diabetic patients aged ≥ 40 years old and with established cardiovascular disease or multiple CV risk factors in a few years^19^.

One impactful question that these data inevitably raise is whether *SGLT* inhibition can confer similar beneficial effects in other cardiac conditions in the absence of diabetes mellitus. The prerequisite for such pharmacological intervention is the upregulation of myocardial *SGLT1* or *SGLT2* as well as intracellular calcium overload in cardiomyocytes. As mentioned previously, other than diabetes mellitus, myocardial *SGLT* protein level is also increased in the presence of myocardial infarction and heart failure^1^ that are associated with intracellular calcium overload. In fact, manipulation of calcium homeostasis of cardiomyocytes, including pharmacological inhibition of sodium entry to lower abnormally high cytosolic calcium concentration, has been recognized as a promising therapeutic strategy for heart failure^17^. Outcome trials to evaluate empagliflozin for the treatment of chronic heart failure amongst patients with and without diabetes mellitus have been planned. The results of these studies might revolutionize our clinical management of heart failure.

Although the EMPA-REG trial has unequivocally demonstrated an early and substantial reduction in non-atherothrombotic cardiovascular events by empagliflozin, the mechanisms responsible for this most astonishing result remain unexplained. The beneficial effects of empagliflozin in reducing the endothelial dysfunction in diabetic mice have been documented recently^3^. In our study using a hiPSC-derived cardiomyocyte-based diabetic cardiomyopathy model, we have provided direct supporting evidence that empagliflozin reverses hyperglycemia-induced hypertrophic changes, alleviates calcium-handling abnormality and restores contractility of high glucose-treated hiPSC-derived cardiomyocytes. This may explain the benefits observed in the clinical trial.

It should be noted that owing to the limitations in patient recruitment, our study was focused on the hiPSCs-derived cardiomyocytes generated from non-diabetic individuals. However, since type 2 diabetes mellitus is a highly heterogeneous disorder that involves complicated interactions between different genetics and environmental factors^20^, the exact effects of any particular drug on different type 2 diabetic patients could vary considerably. In fact, it has also been showed that hiPSC-derived cardiomyocytes generated from different type 2 diabetes patients exhibited different disease phenotypes and differential drug responses^13^. As such, the evaluation of the effects of empagliflozin in hiPSCs generated from different type 2 diabetic patients is anticipated to provide further mechanistically insights into beneficial effects of SGLT inhibitions.

## Methods

### Human iPSC-derived cardiomyocyte-based diabetic cardiomyopathy model

The study protocol for procurement of human tissue for the generation of hiPSCs was approved by the Institutional Review Board of the University of Hong Kong/Hospital Authority Hong Kong West Cluster (HKU/HA HKW IRB) and was registered at the Clinical Trial Center, the University of Hong Kong (HKCTR-725, http://www.hkclinicaltrials.com). Voluntary prior written informed consent was obtained from all participants. All methods were carried out in accordance with the relevant guidelines and regulations provided by the HKU/HA HKW IRB and were registered at the Clinical Trial Center, the University of Hong Kong. In this study, two lines of hiPSCs were used. One hiPSC-line was derived from a healthy Chinese man (Line KS1) with no history or signs of cardiac dysfunction and has been described in our previous publications^21^. Detailed methods of hiPSC generation and characterization, and *in vitro* cardiac differentiation have been previously reported^21–23^. The second hiPSC-line (line IMR90, female) was purchased from WiCell (Madison, Wisconsin, USA). To recreate the chemical environment of diabetes mellitus, hiPSC-derived cardiomyocytes were cultured for 14 days in a medium containing 22 mM glucose, high glucose (HG), in contrast to the standard glucose concentration (NG), 5.5 mM. To test the potential therapeutic effects of empagliflozin on hiPSC-derived cardiomyocytes, the culture medium was supplemented with 5 μM empagliflozin (Selleckchem, TX, USA) at different time points. For the details, please refer to Fig. 2A.

### Immunostaining analysis and cell size measurement

The hiPSC-derived cardiomyocytes were seeded on glass coverslips (12 mm) and cultured in different concentrations of glucose in the presence or absence 5 μM empagliflozin. After treatment, cells were fixed in 2% paraformaldehyde for 10 min at room temperature. After fixation, cells were permeabilised with a wash buffer (1X PBS containing 0.1% Triton-X 100). Afterwards, the coverslips were incubated with a blocking buffer (wash buffer with 5% normal donkey serum) for 1 hour at room temperature and subsequently incubated with a mouse monoclonal antibody specific to sarcomeric α-Actinin (A7811, Sigma) in a 1:1000 dilution overnight at 4 °C. Following three washes in wash buffer, the coverslips were incubated with Alexa Fluor 647 conjugated secondary antibodies (Molecular probes) for 1 hr at room temperature. Free secondary antibodies were removed by three washes in wash buffer, and the coverslips mounted on glass slides with Prolong gold anti-fade medium (containing DAPI) (Molecular probes) and examined under standard fluorescent microscopy. Cell size was measured using Image J software (imagej.nih.gov).

### Measurements of contractility and intracellular calcium transient

Contractility and intracellular calcium transient of hiPSC-derived cardiomyocytes were evaluated with the IonOptix myocyte calcium and contractility system (IonOptix, Milton, MA)^24^. Briefly, hiPSC-derived cardiomyocytes cultured on gelatin-coated coverslips were loaded with 1 μM fura-2-AM (Sigma-Aldrich, St. Louis, MO), a calcium-sensitive, radiometric fluorescence dye for 15 min, and then transferred to a perfusion chamber mounted on the stage of an inverted microscope (Olympus, IX-51). The cells were then field stimulated via external platinum electrodes using the MyoPacer stimulator (Ionoptix, MA, USA) at 10 V and 1–4 Hz. Contraction of hiPSC-derived cardiomyocytes was assessed using a video-based edge detection system (IonOptix, MA, USA) to continuously measure the movement of a selected point of the hiPSC-derived cardiomyocytes at 240 Hz. The intracellular calcium transient (A340/380 ratio) was recorded with the MyoCam-S camera (Ionoptix, MA, USA) fixed to the inverted microscope. Data acquisition and analysis were performed using the IonWizard 6.3 software (IonOptix, Milton, MA). The indices measured included peak shortening, time-to-peak shortening, time-to-90% re-lengthening, and maximal velocity of shortening/re-lengthening (±dL/dt).

### Measurement of Extracellular Acidification Rate by Seahorse XF^e^24 Analyzer

Glycolysis in the hiPSC-derived cardiomyocytes was assayed by measuring the extracellular acidification rates (ECARs) in real-time using a XF^e^24 Extracellular Flux Analyzer (Seahorse Bioscience, North Billerica, MA, USA) according to the manufacturer’s protocols. In brief, hiPSC-derived cardiomyocytes (5.0 × 10^4^ cells per well) were seeded onto XF^e^24 cell culture plates (Seahorse Bioscience, North Billerica, MA, USA) and cultured for 24 hours to allow recovery. On the day of measurement, unattached cells (about 5% of the total cell seeded) were washed out with 1x PBS, and the media replaced with XF assay medium (Seahorse Bioscience, North Billerica, MA, USA) supplemented with 5 mM glucose and 1 mM sodium pyruvate. Cells were then incubated at 37 °C without CO_2_ for 1 hour. Stock solutions of glucose, oligomycin and 2-deoxy-glucose (2-DG) were prepared in complete XF assay medium and loaded into injection ports A, B and C, respectively. The injection ports were loaded with optimized concentrations of 10 mM glucose, 1 µM oligomycin and 50 mM 2-DG. The machine was calibrated and the assay performed using the glycolytic stress test assay protocol as suggested by the manufacturer.

### Reverse Transcription Quantitative Polymerase Chain Reaction (RT-qPCR)

Total RNA from hiPSC-cardiomyocytes was extracted with TRI reagent (Life Technologies). Reverse transcription was then performed using 1 µg RNA in a final volume of 20 µl, using a QuantiTect reverse transcription kit (Qiagen, Hilden, Germany) according to the manufacturer’s instructions. Quantitative PCR analysis was performed with a real-time PCR system (StepOne Plus Real Time PCR systems, Applied Biosystems) using the Fast SYBR Green Reagent (Qiagen). The primer sequences for the target genes are listed in Supplementary Table 1. For amplification, after initial holds for 5 min at 95 °C, 40 cycles of 95 °C for 30 seconds followed by corresponding annealing temperature for 30 seconds and 72 °C for 30 seconds, melt curve analysis was performed. The relative quantification of PCR products was performed according to the 2^−ΔΔCt^ method, with *TNNT2* as internal control. Where ΔΔCt = [(Ct_target gene_ − Ct_*TNNT2*_)_Treatment group_ − (Ct_target gene_ − Ct_*TNNT2*_)_Control group]_].

### Western Blot analysis

Cells of interest were harvested by centrifugation and lysed in a RIPA buffer (Thermo Scientific) in the presence of proteinase inhibitor cocktails (Thermo Scientific). Insoluble cell debris was removed by centrifugation and the supernatants saved for analysis. The amount of proteins was quantified using the Bradford reagent (Bio-Rad). For each sample, about 40 µg of total protein was resolved on a pre-cast 4–12% polyacrylamide gel (Invitrogen) and transferred electrically onto a PVDF membrane. The membrane was then incubated with a blocking solution (1XTBS, 0.1% tween-20 and 5% non-fat powdered milk) for 1 hr at room temperature. After blocking, the membrane was incubated with the primary antibodies against *SGLT1* (PA5-28240, invitrogen), *SGLT2* (ab37296, ABCAM), phospholamban (MA3-922, Invitrogen), phopho-phospholamban (phospho S16, ab15000,ABCAM), Caspase 3 (#9662, Cell Signaling TECHNOLOGY), Caspase 7 (#12827, Cell Signaling TECHNOLOGY) or β-actin (sc-130300, Santa Cruz Biotechnology) overnight at 4 °C. After three washes in wash buffer (1X TBS, 0.1% tween-20), the membrane was incubated with HRP-conjugated secondary antibodies for 1 hr at room temperature. Afterwards, the membrane was washed in the wash buffer three times, and the target protein-antibody complexes visualized using standard enhanced chemiluminescence methods.

### Statistical analysis

For immunostaining-based methods, at least 50 fields of cells (about 500 cells) were analyzed for each group. For real time quantitative PCR analysis, data were collected from six repeated experiments. For Western blot analysis, data were collected from three repeated experiments. Continuous variables are expressed as mean ± SEM. Statistical comparisons between two groups were performed using Student’s t test or nonparametric Mann-Whitney test (for sample number smaller than 10). Variables that involved more than two groups were analysed using one-way ANOVA, and Tukey analysis was used as the post-test for comparing the values from all pairs of columns. Calculations were performed with GraphPad Prism 5 (GraphPad). A P value < 0.05 was considered statistically significant.

## Electronic supplementary material

SUPPLEMENTARY INFORMATION
