# Supplementary material for: Empagliflozin Ammeliorates High Glucose Induced-Cardiac Dysfuntion in Human iPSC-Derived Cardiomyocytes
Source: Sci Rep. 2018 Oct 5;8:14872. doi: 10.1038/s41598-018-33293-2 (PMC6173708; doi:10.1038/s41598-018-33293-2)
Supplement: Supplementary file 1 — SUPPLEMENTARY INFORMATION [file 41598_2018_33293_MOESM1_ESM.pdf]

EMPAGLIFLOZIN AMMELIORATES HIGH GLUCOSE INDUCED-CARDIAC DYSFUNTION IN HUMAN IPSC-DERIVED CARDIOMYOCYTES

Kwong-Man NG, PhD; Yee-Man LAU, PhD; Vidhu DHANDHANIA, BSc; Zhujun CAI, BSc; Yee-Ki LEE, PhD; Wing-Hon LAI, PhD; Hung-Fat TSE, MD, PhD and Chung-Wah SIU, MD

Supplementary information

Supplementary Figure S1

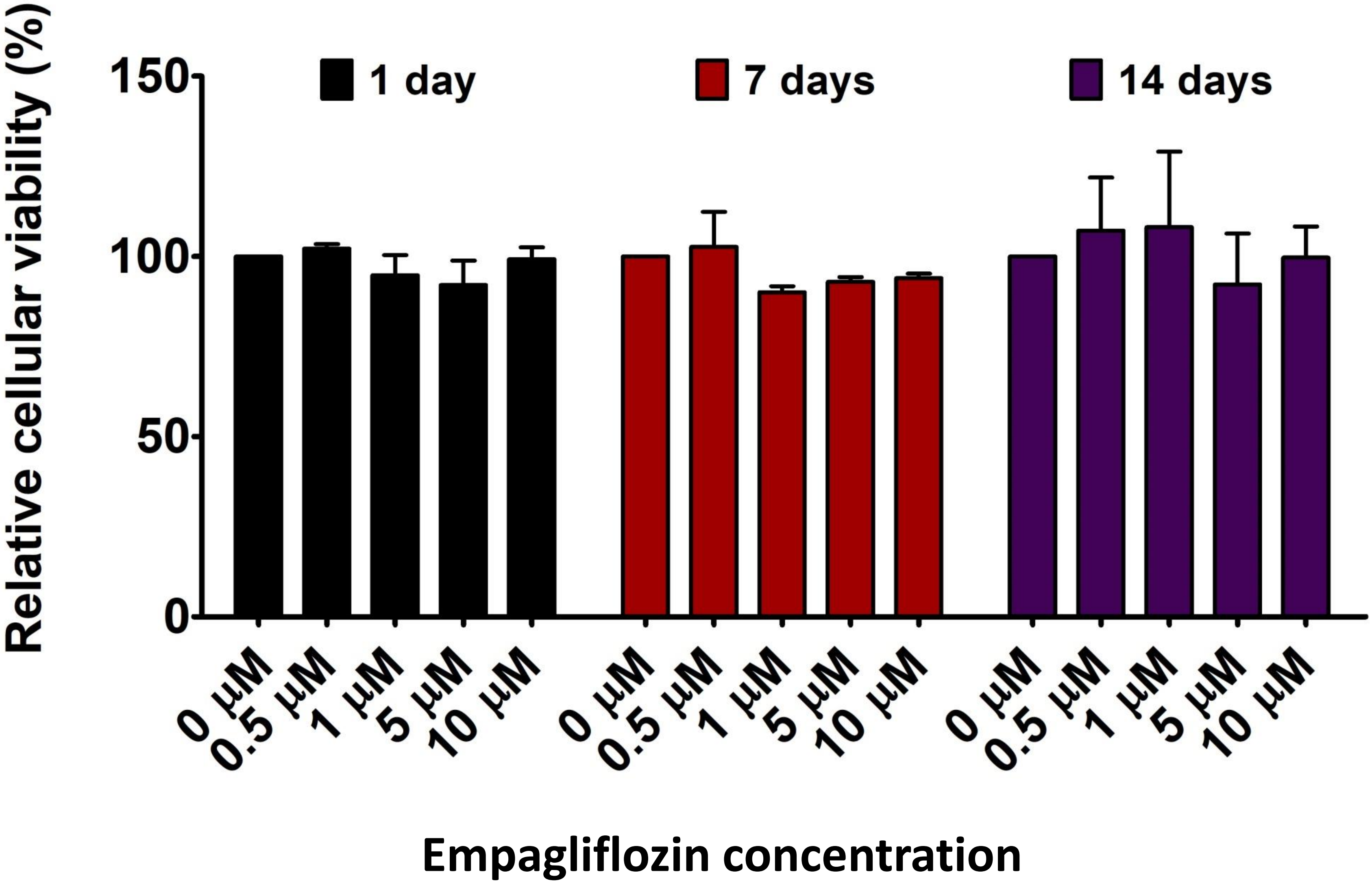

Supplementary Figure S2

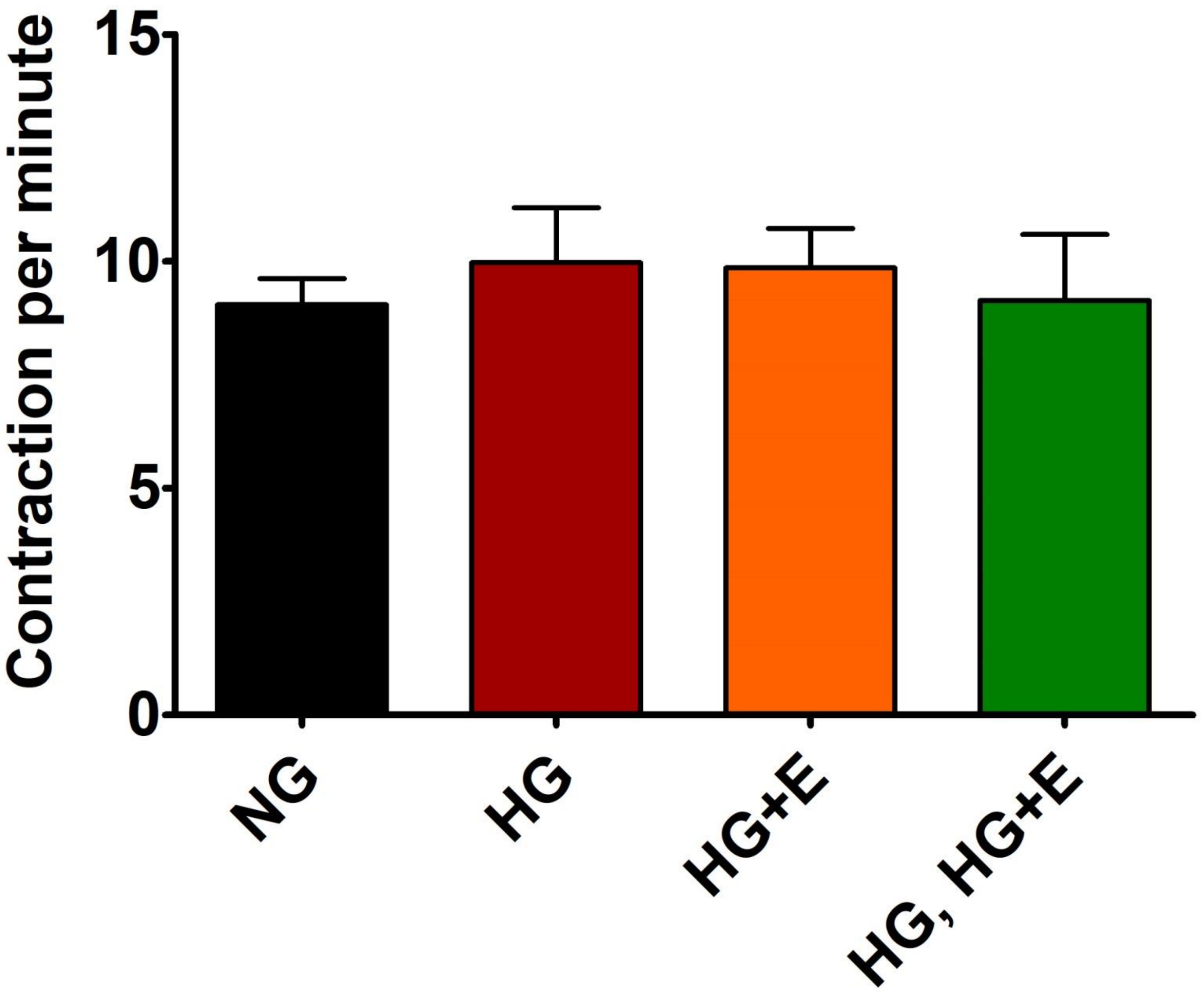

Supplementary Figure S3

A

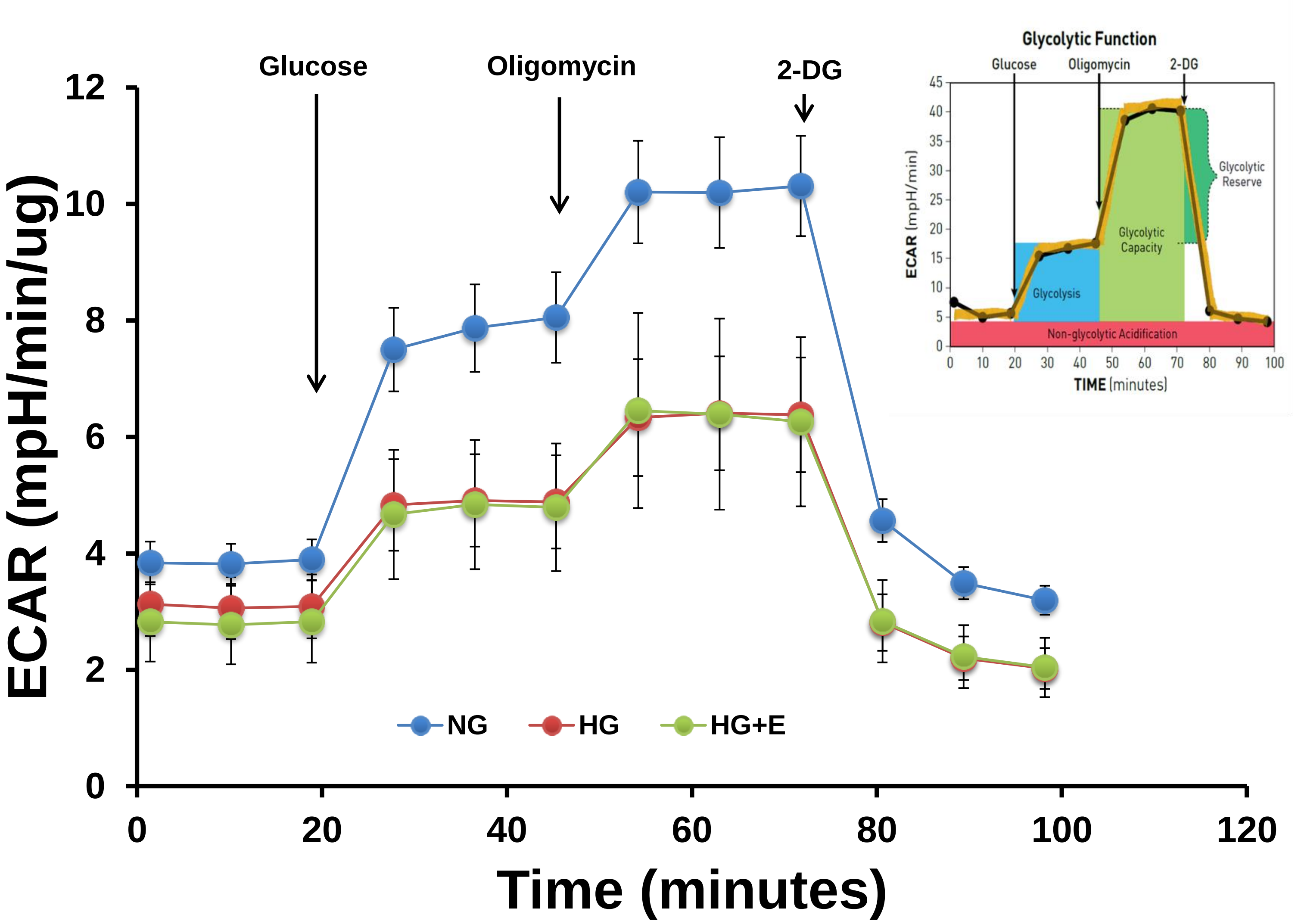

Supplementary Figure S3

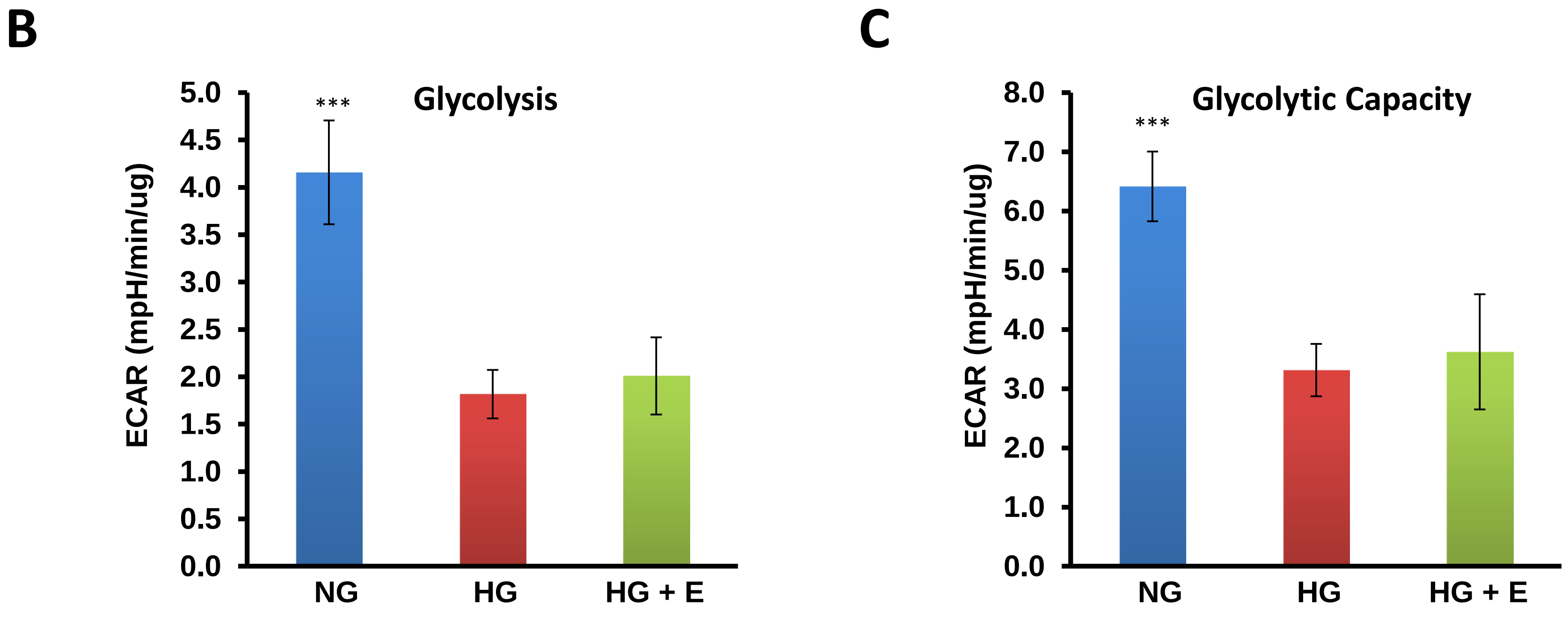

Supplementary Figure S4

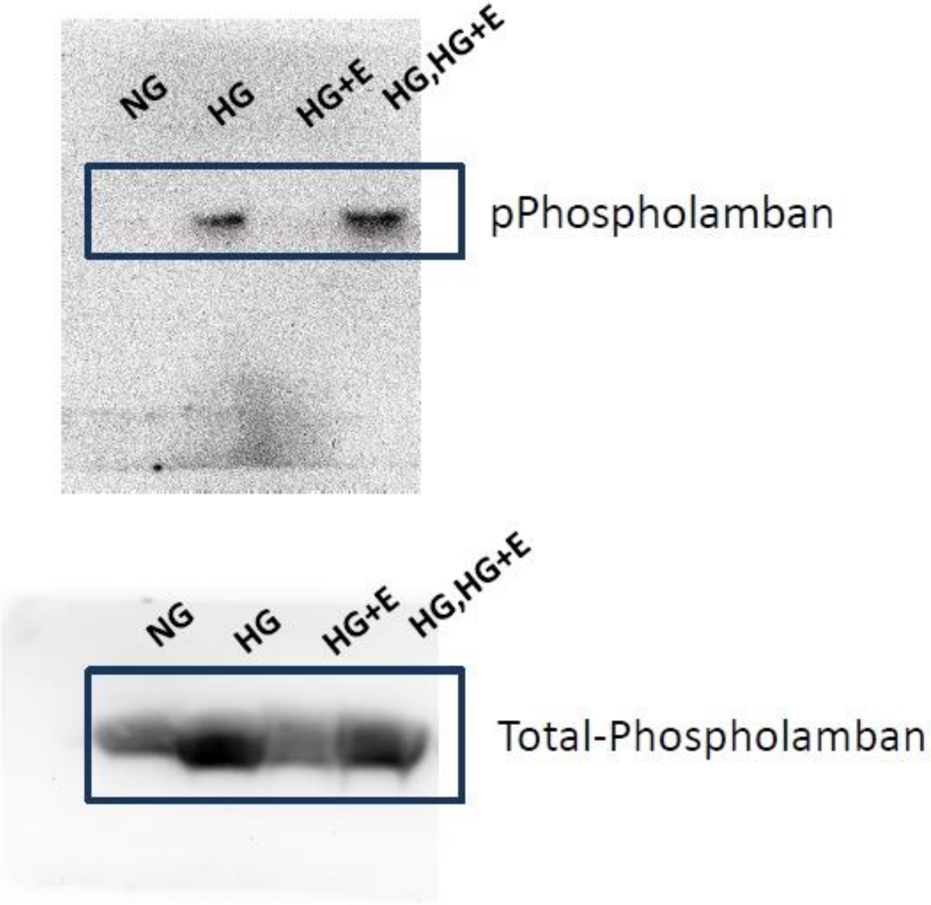

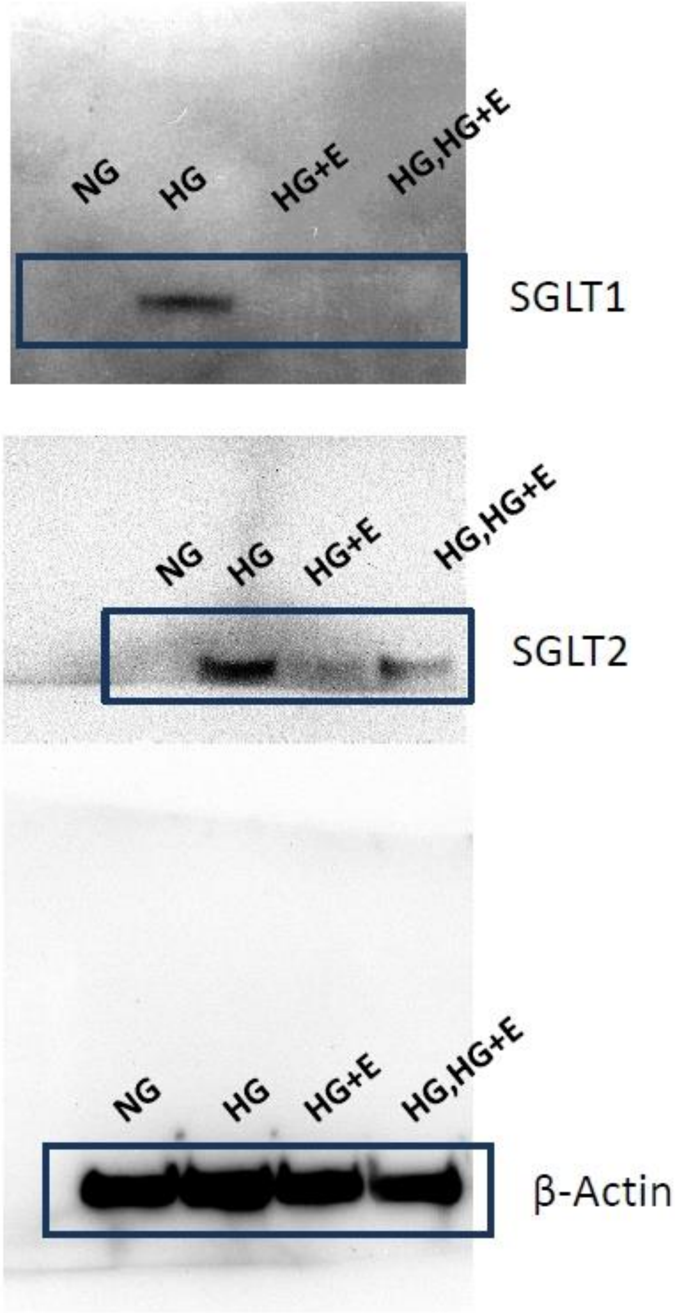

Original Blots of the Western Blot images

Supplementary Figure S5

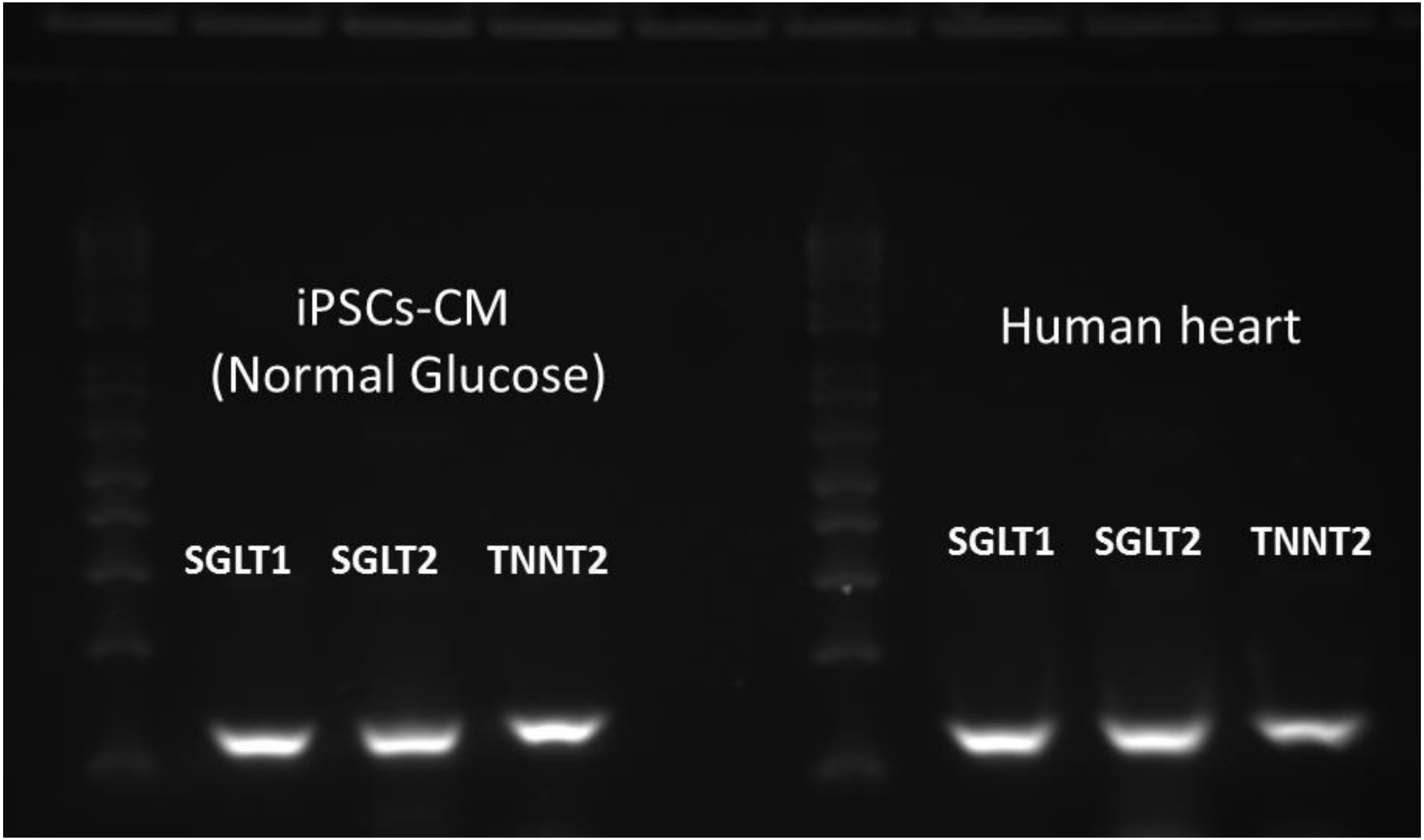

Full gel picture for Figure 6B

Supplementary Table S1. Oligonucleotide primers used in quantitative PCR analysis

Gene of interest	Other name	Primers Sequences
GLUT1	*SLC2A1*	F: ATTCCCAAGTGTGAGTCGCC<br>R: TTGCTGGCTGGAGAAAGGAG
GLUT2	*SLC2A2*	F: CATTGCGGACTTCTGTGGAC<br>R: GCTGAGCCACTCTTCTTTTGG
GLUT3	*SLC2A3*	F: GTCAATGTGCAGTGTAGCCC<br>R: AAGGGAAAGGGAGACTGAGC
GLUT4	*SLC2A4*	F: CTTCCTTTCCTCTGCAGCAC<br>R: AGTCACACGAGGGGAATGAG
SGLT1	*SCL5A1*	F: TCTACATCCCATCCCCATTC<br>R: GAGGAACTTGGTGGTCATGG
SGLT2	*SCL5A2*	F: ACCATAAGCCACAGCCTCAC<br>R: ACTGCCAATCAGATGCAGTG
TNNT2		F: GAATGAAGATCAGCTGAG<br>R: TTATCGTTGATCCTGTTTC
RYR2		F: CGTTCTAACCAGCATCTCATC<br>R: CGAGCAATACAACCTGACC
ATP2A2		F: ACCCACATTCGAGTTGGAAG<br>R: CAGTGGGTTGTCATGAGTGG
NCX1		F: TGTGCATCTCAGCAATGTCA<br>R: TGATGCCAATGCTCTCACTC
ACTA1		F: AGAGCTACGAGCTGCCAGAC<br>R: CGACTCCATACCGATGAAGG
MLC2a		F: GGAGAAGCTCAATGGGACAG<br>R: CCACCTCAGCTGGAGAGAAC
FHL		F: AGTGTGGTGGCCTATGAAGG<br>R: GCTCCTGGTGGAAAACAAAG

Supplementary Figure Legends

Supplementary Figure S1. The cellular viabilities were evaluated using the Cell Counting Kit-8Cell Proliferation / Cytotoxicity Assay Kit (Dojindo molecular technology inc. Maryland, USA). The cellular viability of the untreated control group (0µM empagliflozin) was set to 100% and was used as a reference for comparison.

Supplementary Figure S2. Contraction rates of cardiomyocytes derived from KS1 hiPSCs.

Supplementary Figure S3. Effects of High Glucose and Empagliflozin on Bioenergetics of hiPSC-Derived Cardiomyocytes. (A) Representative Seahorse profiles of hiPSC-derived cardiomyocytes for glycolytic stress testing. Real-time trace of the extracellular acidification rate (ECAR) was determined with a Seahorse XFe24 analyzer. After baseline measurements, cells were subsequently treated with 10 mM glucose, 1 µM oligomycin and 50 mM 2-deoxyglucose (2-DG). The inset shows the schematic of glycolysis stress test. ECAR following the addition of glucose defines glycolysis and ECAR following oligomycin represents the maximum glycolytic capacity. ECAR following treatment with 2-DG represents acidification associated with non-glycolytic activity. (B) The bar charts show the calculated glycolysis and (C) glycolytic capacity. Each data point represents mean ± SEM, n=3. Abbreviations: NG: normal glucose (5.5mM); HG: high glucose (22 mM); and HG+E: high glucose and empagliflozin. ***: $p<0.001$

Supplementary Figure S4. Original blots of Western Blot images.

Supplementary Figure S5. Full gel picture of PCR analysis images

Supplementary Table S1. List of oligonucleotide primers used in the quantitative PCR analysis.
